# Pioglitazone Improved Insulin Sensitivity and First Phase Insulin Secretion Among Obese and Lean People with Diabetes: A Multicenter Clamp Study

**DOI:** 10.1007/s13300-018-0401-9

**Published:** 2018-03-13

**Authors:** Xin Qian, Hui Wang, Gangyi Yang, Zhengnan Gao, Yong Luo, Aimei Dong, Fang Zhang, Mingtong Xu, Shiping Liu, Xin Yang, Yanyan Chen, Guangwei Li

**Affiliations:** 1 Endocrinology and Cardiovascular Diseases Center, Fuwai Hospital, Chinese Academy of Medical Sciences, No. 167 North Lishi Road, Xicheng District, Beijing, China; 2 Department of Endocrinology, The Second Affiliated Hospital, Chongqing Medical University, No. 76 Linjiang Road, Chongqing, China; 3 Department of Endocrinology, Dalian Municipal Central Hospital, No. 42 Xuegong Street, Shahekou District, Dalian, China; 4 Department of Endocrinology, Chongqing Three Gorges Central Hospital, No. 165 Xincheng Road, Wanzhou District, Chongqing, China; 5 Department of Endocrinology, Peking University First Hospital, No. 8 Xishiku Road, Xicheng District, Beijing, China; 6 Department of Endocrinology, Peking University Shenzhen Hospital, No. 1120 Lianhua Road, Futian District, Shenzhen, China; 7 Department of Endocrinology, Second Affiliated Hospital of Sun Yat-sen University, No. 107 Yanjiang West Road, Guangzhou, China; 8 Department of Endocrinology, The Second Xiangya Hospital of Central South University, No. 139 Renmin Middle Road, Changsha, Hunan China; 9 Department of Information Systems, Statistics, and Management Science, Culverhouse College of Commerce and Business Administration, The University of Alabama, Tuscaloosa, AL USA

**Keywords:** First phase, Insulin resistance, Insulin secretion, Pioglitazone

## Abstract

**Introduction:**

To investigate the effects of pioglitazone (PIO) on insulin resistance and first phase insulin secretion among obese and lean Chinese people with type 2 diabetes mellitus (T2DM).

**Methods:**

Sixty-eight drug-naïve patients with T2DM were treated with PIO for 16 weeks. Before and after the treatment, insulin sensitivity was evaluated by the euglycemic hyperinsulinemic clamp test. Plasma insulin levels at 0, 3, 5, 7, and 10 min during intravenous glucose tolerance test were determined to calculate the first phase insulin secretion and pancreatic β-cell function. Circulating adiponectin levels were quantified.

**Results:**

In both the lean and the obese patients with T2DM, the reduction of HbA_1c_ following the PIO treatment was more than 1% (*P *< 0.001) and glucose infusion rate, acute insulin response, glucose disposal index, and β-cell glucose sensitivity increased significantly (*P *< 0.001). A multiple linear regression analysis showed that the improvements of first phase insulin secretion and insulin sensitivity were independently associated with the changes of HbA_1c_, but the change of first phase insulin secretion exhibited a higher correlation coefficient (*R*^2^ = 0.20, *P *= 0.001) than the change of insulin sensitivity did (*R*^2^ = 0.07, *P *= 0.040). The PIO treatment led to a significant increase in adiponectin levels only in the obese group (*P *< 0.05).

**Conclusion:**

A 16-week treatment of PIO significantly increased insulin sensitivity and β-cell function in the lean group as well as in the obese group among Chinese T2DM patients, demonstrating that both lean and obese diabetic adults would profit from PIO.

**Trial registration:**

The ChiCTR registry number is ChiCTR-OPC-17011571.

**Funding:**

Takeda Pharmaceutical Co. Ltd. and Pfizer Pharmaceutical Co. Ltd.

## Introduction

It is believed that obesity is a marker of insulin resistance which exists in the majority of patients with type 2 diabetes mellitus (T2DM) [1–3]. How about the insulin sensitivity in non-obese persons with type 2 diabetes? This is particularly important for the Asian diabetic population since obesity was not as prevalent among Asians as in western countries [1, 4, 5]. Furthermore, a practical question is whether insulin secretagogues such as sulfonylureas, rather than insulin sensitizers such as metformin or thiazolidinediones, should be used as first-line antidiabetes medication in non-obese patients with type 2 diabetes. To what extent impaired insulin sensitivity exists in non-obese patients with diabetes remains unclear [4]. Results of our previous pilot study suggested that insulin resistance also existed in the newly diagnosed non-obese Chinese patients with type 2 diabetes and severe hyperglycemia. One explanation is that the development of insulin resistance in this population is induced, at least to some extent, by glucose toxicity. However, to our best knowledge, whether the insulin resistance induced by glucose toxicity can also be overcome by insulin sensitizer administration has not been widely studied. The aim of the present study was to investigate if a 16-week pioglitazone (PIO) therapy can improve first phase insulin secretion and insulin resistance in newly diagnosed non-obese type 2 diabetes patients in comparison with obese diabetes patients.

## Methods

### Patients

We recruited 68 drug-naïve and newly diagnosed diabetic patients (male and female) aged from 25 to 60 years. Thirty-one patients were lean with body mass index (BMI) less than 25 kg/m^2^ and 37 patients were obese with BMI at least 25 kg/m^2^. The patients eligible for enrollment had a fasting plasma glucose (FPG) level no more than 11.1 mmol/l. The patients were excluded if they had type 1 or type 2 diabetes with diabetic ketoacidosis (DKA), infection and other stress status, autoimmune disease, hepatic and renal diseases, severe heart failure (NYHA III and IV), symptomatic heart failure, established edema, or increased risk of fractures.

### Study Design

Eight hospitals in six provinces and cities in China participated in the study. Euglycemic hyperinsulinemic clamp test was used to evaluate the status of insulin resistance. The acute insulin response (AIR) was adopted to determine β-cell function of first phase insulin secretion. In order to investigate the mechanism of glucose reduction, the changes of insulin resistance and pancreatic β-cell function were analyzed after normalization of glucose control which was achieved by the PIO therapy. Medications other than PIO such as those for controlling dyslipidemia and hypertension remained unchanged during entire the trial. Participants were asked to maintain their usual lifestyles including diet and physical activities throughout the study.

All procedures followed were in accordance with the ethical standards of the responsible committee on human experimentation (institutional and national) and with the 1964 Declaration of Helsinki, as revised in 2013. This study was approved by the Ethics Committee of Fuwai Hospital Chinese Academy of Medical Sciences. All patients gave written informed consent prior to data collection.

### Study Procedures

Patients were required to fast overnight before undergoing an intravenous glucose tolerance test (IVGTT) the next morning. Blood samples were collected at 0, 3, 5, 7, and 10 min for the measurements of glucose and insulin. Glycated hemoglobin (HbA_1c_), hepatic and renal function, lipid profile, and urine tests were also examined under the fasting status. After that, the euglycemic hyperinsulinemic clamp test was performed on the same day. Insulin (Novolin R; NovoNordisk, Bagsvaerd, Denmark) in normal saline solution and 20% glucose solution were infused separately into the ulnar veins. The dorsum manus vein was used for extracting arterialized venous blood after warming the arm to 50–55 °C with an infrared heater. The insulin infusion rate was adjusted according to the target plasma glucose level and maintained at 1 mU/kg/min for the next 120 min, and blood was drawn every 5 min to measure plasma glucose concentration. Adjustment of the infusion rate of 20% glucose solution was made to keep the blood glucose approximately at the target value, 5.0 mmol/L. The mean glucose infusion rate (GIR) value was estimated to be equal to the GIR in the last 30 min of steady state. Insulin in the plasma samples was analyzed using a immunoradiometric assay (IRMA, CIS bio international, Bagnols, France). Additional baseline assessments included blood pressure, height, and body weight.

PIO treatment was initiated after the completion of the tests described above. A dose of 30 mg per day was administered initially and increased to 45 mg if needed. The duration of treatment was 16 weeks. The patients were followed monthly and PIO dosages were adjusted to achieve the target goal of plasma glucose control (FPG < 6.1 mmol/L and 2-h plasma glucose level, 2hPG < 7.8 mmol/L). During each follow-up visit, levels of FPG and 2hPG were measured, and urinalysis was performed. All measurements at baseline, including the IVGTT and the euglycemic hyperinsulinemic clamp test, were repeated at the end of the 16-week PIO treatment.

### Measurements and Calculations

Insulin sensitivity was evaluated by GIR (mg/kg/min) based on the euglycemic hyperinsulinemic clamp test. The area under the curve of insulin during IVGTT was quantified as first phase insulin secretion (AIR, min × µU/ml). Glucose disposal index (DI), the products of GIR and AIR, was also calculated. Ratios of the area under the curve of insulin (AUC-Insulin) to the area under the curve of glucose (AUC-Glucose, Ratio AUC-I/G) were calculated to evaluate β-cell sensitivity to glucose. Plasma levels of leptin, adiponectin (APN), and high sensitivity C-reactive protein (hs-CRP) were also measured at baseline and at the end of the 16-week PIO treatment.

### Statistical Analysis

Mean value and standard deviation (SD) were reported for continuous variables. Number and percentage (%) were used for categorical variables. For right-skewed distribution variables, a median, 25 percentile and 75 percentile of the measure were reported. The changes of insulin sensitivity, plasma insulin, and glucose DI levels from baseline to the end of PIO treatment in each subgroup (the obese group and the lean group) were analyzed by pairwise *t* tests. A mixed model for repeated measures (MMRM) analysis was used to compare the differences between the lean and the obese group. For variables not normally distributed, npar1way analysis was performed to find the differences between the lean and the obese group. The adverse events related to the study were assessed.

## Results

### Reduction of HbA_1c_ and Changes of Insulin Sensitivity

**Fig. 1:**
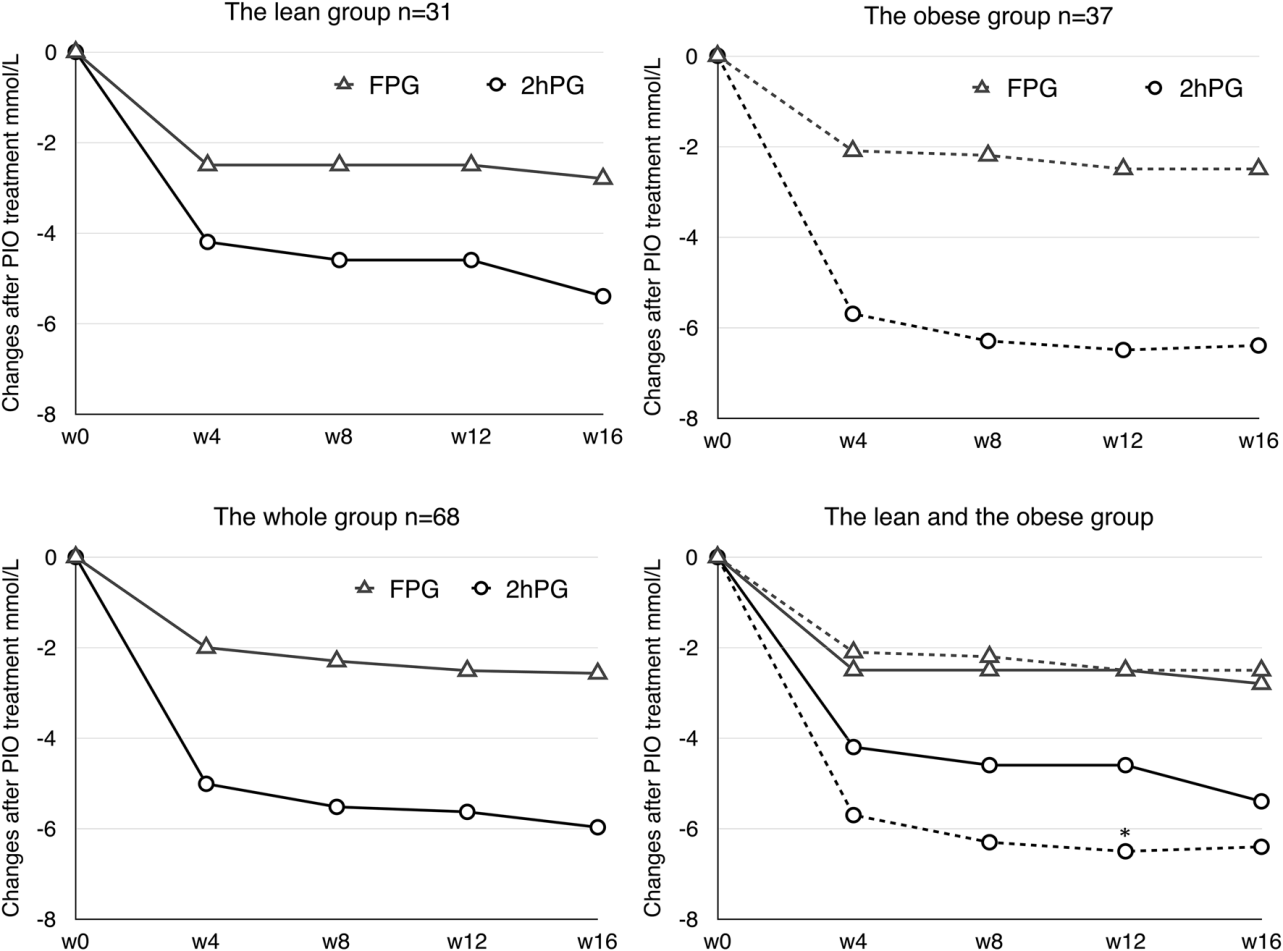
Plasma glucose changes after PIO treatment in the lean, obese, and whole groups. *P < 0.01 compare to the lean group

**Fig. 2:**
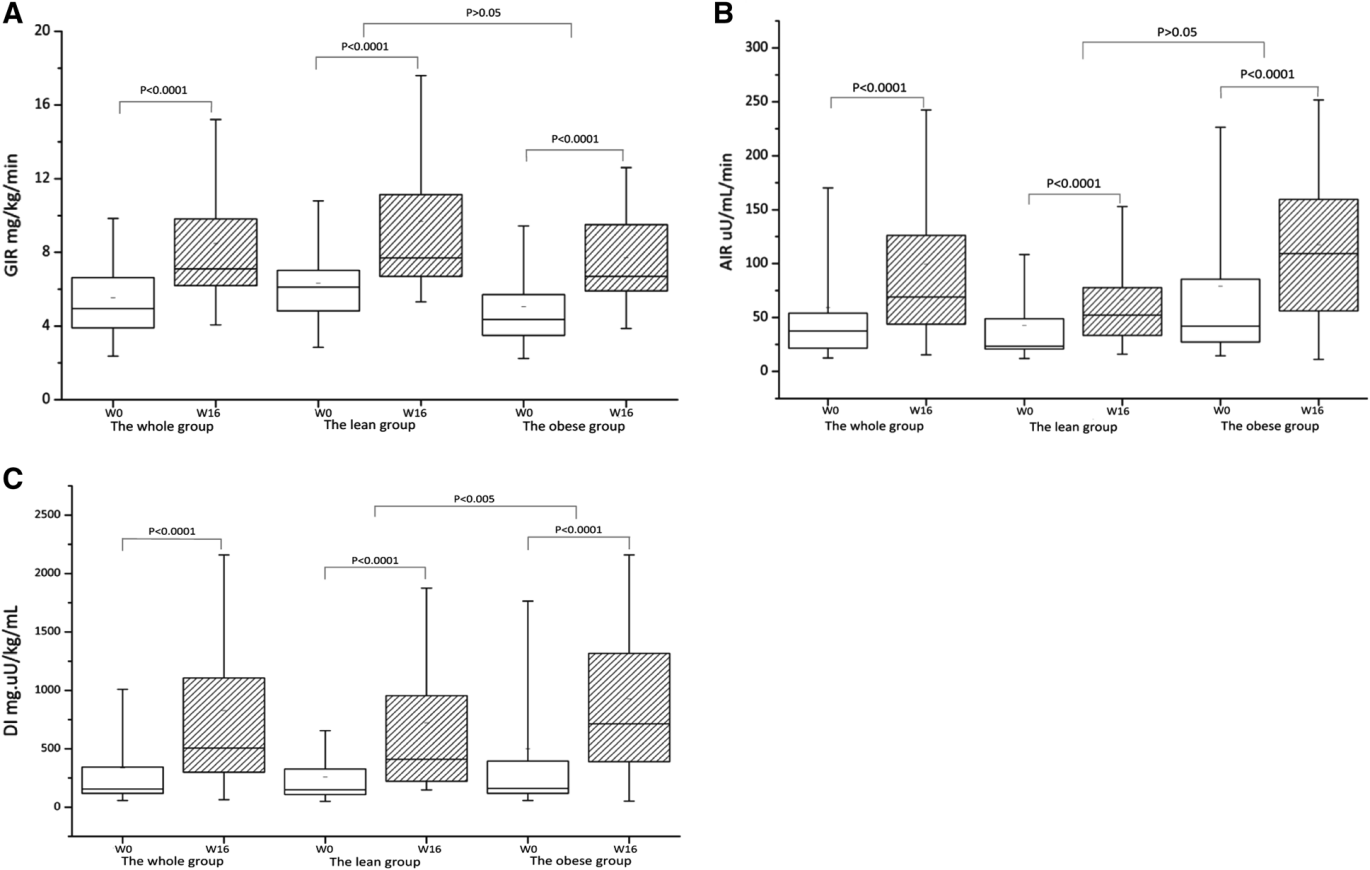
Glucose infusion rate (GIR), acute insulin response (AIR), and glucose disposal index (DI) before and after PIO treatment in the whole, lean, and obese groups. DI, the products of GIR and AIR

Sixty-eight newly diagnosed diabetic patients, comprising 31 persons in the lean group (BMI 22.5 ± 1.6 kg/m^2^) and 37 persons in the obese group (BMI 28.3 ± 2.8 kg/m^2^), participated in and completed the 16-week trial. The dose of PIO was 30 mg per day initially in both subgroups, and increased to 40 mg on average in the lean group and 35 mg on average in the obese group from 4 to 16 weeks. After the 16-week treatment, PIO decreased HbA_1c_ by 1.3 ± 1.1% in whole newly diagnosed T2DM patients with a mean baseline level of HbA_1c_ at 7.6 ± 1.2%, resulting in a mean HbA_1c_ level less than 6.5% (6.4 ± 0.6%) at the end of the trial. Accordingly, the mean FPG and 2hPG values in all patients were reduced by 2.7 ± 2.0 mmol/L and 6.8 ± 0.5 mmol/L, respectively. The mixed model analysis showed that only 2hPG change at 12 weeks from baseline was significantly different between the two subgroups over time (*P *< 0.01) (Fig. 1). But the reduction of HbA_1c_ induced by PIO was not significantly different between the lean and the obese group (1.2 ± 0.2 vs. 1.3 ± 0.2%, *P *> 0.05) (Table 1). The median of insulin-mediated glucose disposal rate during the euglycemic hyperinsulinemic clamp test (by GIR) was 5.0 mg/kg/min in the whole study group, 6.1 mg/kg/min in the lean group, and 4.4 mg/kg/min in the obese group, respectively, at baseline. After the 16-week PIO treatment, the median of GIR increased to 7.1 mg/kg/min in the whole group, 7.7 mg/kg/min in the lean group, and 6.7 mg/kg/min in the obese group, respectively. The changes of GIR in the whole group and both the lean and the obese group were significantly different (*P *< 0.0001) but not statistically different between the lean and the obese group (*P *> 0.05) (Table 1, Fig. 2a).

**Table 1:** Summary of efficacy parameters of a 16-week PIO treatment in the whole, lean, and obese groups of type 2 diabetes patients

	Whole group (*n* = 68)			Lean group (*n* = 31)			Obese group (*n* = 37)		
	Baseline	16th week	Changes	Baseline	16th week	Changes	Baseline	16th week	Changes
HbA_1c_ (%)	7.6 ± 1.2	6.4 ± 0.6	− 1.3 ± 1.1^a^	7.7 ± 1.0	6.4 ± 0.5	− 1.2 ± 0.2^a^	7.6 ± 1.3	6.4 ± 0.6	− 1.3 ± 0.2^a^
FPG (mmol/L)	8.6 ± 1.9	6.1 ± 0.7	− 2.7 ± 2.0^a^	8.7 ± 2.0	5.9 ± 0.7	− 3.0 ± 0.4^a^	8.6 ± 1.8	6.1 ± 0.7	− 2.5 ± 0.4^a^
2hPG (mmol/L)	13.8 ± 2.9	7.8 ± 1.5	− 6.8 ± 0.5^a^	13.1 ± 2.6	7.7 ± 1.6	− 6.0 ± 0.6^a^	14.3 ± 3.1	7.9 ± 1.3	− 7.7 ± 0.6^a^
GIR (mg/kg/min)	5.0 (3.9–6.6)	7.1 (6.2–9.8)	2.0 (0.9–3.8)^a^	6.1 (4.8–7.0)	7.7 (6.7–11.1)	1.5 (0.8–4.4)^a^	4.4 (3.5–5.7)	6.7 (5.9–9.5)	2.4 (1.6–3.7)^a^
AIR (µU/ml/min)	37.5 (21.5–54.1)	69.1 (43.8–126.1)	28.3 (11.1–65.7)^a^	23.4 (20.9–48.8)	52.2 (33.4–77.7)	17.6 (8.9–41.1)^a^	42.0 (27.3–85.5)	109.2 (56.0–159.6)	57.5 (27.1–90.8)^a^
DI (mg × µU/kg/mL)	155.3 (118.5–343.9)	506.7 (299.8–1105.4)	305.4 (157.0–746.1)^a^	149.8 (107.6–327.5)	411.1 (221.0–955.2)	164.4 (85.2–433.4)^a^	160.6 (118.5–394.0)	712.8 (389.8–1316.9)	472.5 (297.9–1108.1)^a,d^
Ratio AUC-I/G	0.3 (0.1–0.5)	0.5 (0.3–1.0)	0.2 (0.1–0.5)^b^	0.2 (0.1− 0.3)	0.4 (0.2–0.6)	0.2 (0.1–0.3)^b^	0.4 (0.2–0.7)	0.9 (0.4–1.4)	0.3 (0.1–0.6)^b^
Leptin (ng/mL)	2.4 (1.1–6.6)	2.8 (1.0–8.5)	0.2 (− 1.54 to 2.7)	1.9 (0.9–5.4)	2.6 (0.8–8.5)	1.0 (− 1.7 to 3.5)	3.0 (1.6–7.8)	2.4 (0.7–6.8)	− 0.7 (− 1.8 to 1.3)
APN (µg/mL)	9.8 (5.7–21.1)	25.2 (11.3–52.4)	12.6 (− 2.7 to 38.3)^c^	14.1 (8.1–30.3)	28.9 (12.2–58.6)	10.2 (− 14.8 to 40.4)	8.4 (5.0–11.5)	22.3 (10.9–42.5)	12.6 (2.0–32.2)^c^
hs-CRP (ng/mL)	1.8 (1.0–3.0)	1.3 (0.7–2.5)	− 0.3 (− 1.7 to 0.9)	1.8 (1.0–3.3)	1.9 (0.7–2.8)	− 0.3 (− 1.5 to 1.1)	1.8 (1.2–2.9)	1.3 (0.7–2.4)	− 0.3 (− 1.7 to 0.6)

Data are mean ± standard deviation or median (interquartile range, IQR)

*GIR* glucose infusion rate, *AIR* acute insulin response, DI (Glucose disposal index) = GIR × AIR, *Ratio AUC-I/G* area under curve of insulin/area under curve of glucose

^a^*P *< 0.001

^b^*P *< 0.01

^c^*P *< 0.05, compared to baseline

^d^*P *< 0.01, compared to lean group

### Improvements of Pancreatic β-Cell Function

The median (interquartile range, IQR) of AIR after 75 g intravenous glucose load was 37.5 (21.5–54.1) µU/ml/min in the whole study group, 23.4 (20.9–48.8) µU/ml/min in the lean group, and 42.0 (27.3–85.5) µU/ml/min in the obese group, respectively, at baseline. After the 16-week PIO treatment, the median (IQR) of AIR increased and was 69.1 (43.8–126.1) µU/ml/min in the whole group, 52.2 (33.4–77.7) µU/ml/min in the lean group, and 109.2 (56.0–159.6) µU/ml/min in the obese group, respectively. The changes of AIR were significant in the lean and the obese group (*P *< 0.0001) (Table 1, Fig. 2b). The median (IQR) of glucose DI was improved accordingly: from 155.3 (118.5–343.9) to 506.7 (299.8–1105.4) mg × µU/kg/mL in the whole study group (*P *< 0.0001), from 149.8 (107.6–327.5) to 411.1 (221.0–955.2) mg × µU/kg/mL in the lean group (*P *< 0.001), and from 160.6 (118.5–394.0) to 712.8 (389.8–1316.9) mg × µU/kg/mL in the obese group (*P *< 0.001), respectively. The changes of DI were significantly different between the lean and the obese group (*P *< 0.01) (Table 1, Fig. 2c). Ratio AUC-I/G, an index of glucose sensitivity of β-cells, was increased in the whole sample and both the lean and the obese group. The changes of Ratio AUC-I/G were 0.2 (0.1–0.5), 0.2 (0.1–0.3), and 0.3 (0.1–0.6) in whole sample, the lean group, and the obese group, respectively (*P *< 0.001). The changes of AIR and Ratio AUC-I/G were not statistically different between the lean and the obese group (*P *> 0.05).

### Effects of PIO-Induced Improvement of Insulin Sensitivity and β-Cell Function on Reduction of HbA_1c_

To investigate the effects of PIO-induced improvement of insulin sensitivity (Change_GIR) and β-cell function (Change_AUC-I/G) on reduction of HbA_1c_, a multiple linear regression analysis was performed using change of HbA_1c_ as a dependent variable and changes of insulin sensitivity and insulin secretion induced by PIO treatment as predictors. The results showed that one-SD increases in both GIR and the Ratio AUC-I/G were significantly associated with the reduction of HbA_1c_ (*P* = 0.04 vs. *P* = 0.003, Table 2), and that the Change_ratio AUC-I/G had a more powerful influence on the change of HbA_1c_ than the Change_GIR (partial *R*^2^ = 0.20 vs. partial *R*^2^ = 0.07, Table 2).

**Table 2:** Multiple linear regression analysis using the change of HbA_1c_ as a dependent variable and the change of insulin sensitivity and insulin secretion induced by PIO treatment as predictors

Variable	Estimate	SE	*P* Value
Intercept	0.00521	0.12575	0.9671
Change_GIR	− 0.26663^a^	0.12610	0.0397
Change_Ratio AUC-I/G	− 0.39765^a^	0.12654	0.0029

Variable entered	Partial *R*^2^	Model *R*^2^	*F* Value	*P* Value
Change_GIR	0.0681	0.2688	4.47	0.0397
Change_ratio AUC-I/G	0.2007	0.2007	12.30	0.0010

Dependent variable: change of HbA_1c_ after PIO treatment compared to baseline. The baseline of GIR and ratio AUC-I/G did not enter the model at *P *< 0.05 level

^a^For one-SD increase

### Changes of APN, hs-CRP, and Leptin

PIO increased plasma APN [the median (IQR)] significantly in the total study sample from 9.8 (5.7–21.1) to 25.2 (11.3–52.4) μg/mL (*P *< 0.05) and in the obese group from 8.4 (5.0–11.5) to 22.3 (10.9–42.5) μg/mL, respectively (*P *< 0.05). However, plasma APN was not significantly increased in the lean group with the level from 14.1 (8.1–30.3) to 28.9 (12.2–58.6) μg/mL (*P *> 0.05). In addition, PIO reduced plasma hs-CRP in the whole group and both the lean and the obese group, but the reductions of hs-CRP after treatment were not significant (*P *> 0.05). No significant changes were observed in plasma leptin level after the PIO treatment. The changes of APN, hs-CRP, and leptin were not statistically different between the lean and the obese group (*P *> 0.05).

### Safety and Adverse Events

No severe adverse event was found in the study (Table 3). Blood pressure was decreased, but only the decrease of diastolic blood pressure in the obese group was significant (*P* = 0.01). The changes of body weight and BMI after the 16-week PIO treatment were not significant in the whole sample (69.5 ± 12.2 vs. 69.1 ± 11.8 kg for body weight, 25.6 ± 3.7 vs. 25.5 ± 3.6 kg/m^2^ for BMI, respectively). No significant reduction of triglycerides (TG) and total cholesterol (TC) levels was found in the lean and the obese group (*P *> 0.05). The levels of alanine aminotransferase (ALT) and aspartate aminotransferase (AST) were reduced significantly compare to the baseline in the whole group (*P *< 0.05), but the reduction was not statistically different between the lean and the obese group. Some mild edema (13.2%) and anemia (5.9%) occurred in the whole sample.

**Table 3:** Summary of metabolic and laboratory parameters of a 16-week PIO treatment in the whole, lean, and obese groups of type 2 diabetes patients

	Whole group (*n* = 68)			Lean group (*n* = 31)			Obese group (*n* = 37)		
	Baseline	16th week	Changes	Baseline	16th week	Changes	Baseline	16th week	Changes
BMI (kg/m^2^)	25.6 ± 3.7	25.5 ± 3.6	− 0.2 ± 1.3	22.5 ± 1.6	22.5 ± 2.0	0.04 ± 0.9	28.3 ± 2.8	27.9 ± 2.6	− 0.4 ± 1.5
Weight (kg)	69.5 ± 12.2	69.1 ± 11.8	− 0.6 ± 3.3	60.3 ± 8.5	60.8 ± 9.2	0.1 ± 2.3	77.4 ± 9.0	76.0 ± 9.0	− 1.1 ± 3.9
SBP (mmHg)	124.8 ± 12.9	122.2 ± 12.5	− 2.5 ± 10.9	122.7 ± 14.0	120.7 ± 13.7	− 3.4 ± 11.9	126.6 ± 11.8	123.9 ± 11.3	− 1.7 ± 10.0
DBP (mmHg)	77.3 ± 9.5	74.6 ± 8.4	− 2.7 ± 9.3	75.2 ± 10.4	74.4 ± 7.8	− 1.6 ± 9.8	79.2 ± 8.3	74.5 ± 9.2	− 3.7 ± 8.9^a^
TG (mmol/L)	1.8 ± 1.2	1.7 ± 1.5	− 0.1 ± 1.0	1.5 ± 0.8	1.3 ± 0.6	− 0.3 ± 0.8	2.1 ± 1.4	2.0 ± 1.9	− 0.1 ± 1.3
TC (mmol/L)	4.8 ± 1.0	4.7 ± 0.9	− 0.2 ± 1.1	4.8 ± 1.1	4.6 ± 0.7	− 0.4 ± 1.7	4.9 ± 0.9	4.8 ± 1.1	− 0.0 ± 1.0
HDL-c (mmol/L)	1.1 ± 0.3	1.2 ± 0.3	0.1 ± 0.2	1.1 ± 0.4	1.2 ± 0.3	0.1 ± 0.2	1.1 ± 0.2	1.2 ± 0.3	0.1 ± 0.2
LDL-c (mmol/L)	3.0 ± 0.8	3.0 ± 0.7	− 0.04 ± 0.7	3.0 ± 0.9	2.8 ± 0.5	− 0.1 ± 0.5	3.0 ± 0.7	3.0 ± 0.8	0.0 ± 0.9
ALT (IU/L)	24.7 ± 13.0	16.7 ± 6.6	− 6.5 ± 9.5^a^	21.7 ± 14.3	14.8 ± 5.1	− 4.3 ± 6.1^a^	27.2 ± 11.6	18.2 ± 7.3	− 7.8 ± 11.2^a^
AST (IU/L)	22.6 ± 9.2	18.2 ± 5.5	− 3.4 ± 7.7^a^	21.8 ± 11.4	18.0 ± 4.3	− 1.7 ± 6.1^a^	23.2 ± 7.0	18.4 ± 6.5	− 5.0 ± 8.7^a^
Cr (µmol/L)	67.5 ± 17.2	68.2 ± 17.1	0.9 ± 17.4	65.3 ± 16.1	69.1 ± 18.7	3.0 ± 22.6	69.3 ± 18.0	67.5 ± 16.0	− 0.9 ± 11.8
BUN (mmol/L)	5.2 ± 1.5	5.7 ± 1.4	0.7 ± 1.2	5.5 ± 1.7	5.9 ± 1.5	0.7 ± 1.1	5.0 ± 1.4	5.6 ± 1.3	0.6 ± 1.3
Anemia % (*n*)	0	5.9% (4)		0	9.7% (3)		0	2.7% (1)	
Edema % (*n*)	0	13.2% (9)		0	12.9% (4)		0	13.5% (5)	

*ALT* alanine aminotransferase, *AST* aspartate aminotransferase, *Cr* serum creatinine, *BUN* blood urea nitrogen

^a^*P *< 0.05 compare to baseline

## Discussion

Type 2 diabetes is characterized by hyperglycemia accompanied by systemic resistance to insulin and β-cell dysfunction including abnormality of insulin secretion and sensitivity to glucose [3, 6]. Obesity is an important risk factor for T2DM and insulin resistance [7–9]. Whereas, β-cell dysfunction was considered to be the major defect of non-obese type 2 diabetes patients who had less insulin resistance. Therefore healthcare providers may prefer insulin secretagogues not insulin sensitizers as the first choice in the management of non-obese T2DM patients. Our study is the first one, based on euglycemic hyperinsulinemic clamp tests, to investigate the effects of PIO on insulin resistance and β-cell function in Asian type 2 diabetic patients, in particular those with low BMI (< 25 kg/m^2^).

As expected, PIO significantly increased insulin sensitivity in the obese type 2 diabetic patients who were generally believed to be insulin resistant. This finding was supported by other previous studies which revealed that PIO increased hepatic and peripheral tissue sensitivity to insulin in obese diabetic patients [10–13]. Of note, these studies used clamp or Matsuda insulin sensitivity index (not HOMA model assessments) to evaluate the insulin sensitivity. As for the lean persons with diabetes, the existence of insulin resistance remains controversial. Chiu et al. reported that Asian-Americans are more likely to be insulin resistant despite less obesity [14, 15]. The results of our study showed that Chinese patients in the lean group were also insulin resistant at the beginning when they were first diagnosed with T2DM. Furthermore, a significant improvement of insulin sensitivity was achieved after the 16-week PIO treatment in the lean diabetic Chinese patients. Two studies have described the change of insulin sensitivity after PIO treatment in less obese (but not real lean) diabetes patients. One study found that PIO significantly decreased HOMA-IR [16], but in another study no significant decrease of insulin resistance was found in non-obese patients even though blood glucose was effectively controlled by PIO [17]. It may be that the method used for evaluating insulin sensitivity led to the different findings between the two Japanese studies and our study. A surrogate index (the HOMA-IR) was used in the Japanese studies while a gold standard (clamp test) was used in our study to evaluate insulin resistance. In our study, the first phase insulin secretion was significantly improved in both the lean and the obese group consistent with the improvement of β-cell glucose sensitivity index (Ratio AUC-I/G during IVGTT) after the PIO treatment. The improvements of β-cell function including insulin secretion and glucose sensitivity were in line with the fact that HbA_1c_ level was similarly reduced by more than 1.0% after the treatment in each subgroup (with PIO 40 mg per day in the lean group and with PIO 35 mg per day in the obese group). This was strong evidence to show that the glucose toxicity induced by hyperglycemia greatly contributed to the deterioration of β-cell function in the natural history of diabetes.

The improvements of both insulin sensitivity and insulin secretion induced by PIO were significantly and independently associated with the reduction of HbA_1c_ in the present study (*P* = 0.04 vs. *P* = 0.003). That is to say both of them participate in the glucose control. It raises an important question: what is the exact underling mechanism of insulin sensitizers in improving glucose control? To evaluate the influences of insulin sensitivity and β-cell glucose sensitivity, further step regression analysis was performed. The improvement of the first phase insulin secretion function made a greater contribution towards reducing glucose (partial *R*^2^ = 0.20) than the improvement of insulin sensitivity did (partial *R*^2^ = 0.07). These data suggested that the increased insulin sensitivity induced by the PIO treatment corrected the hyperglycemia and ameliorated glucose toxicity, and then resulted in a restoration of β-cell function. It is worth noting that it was the increased first phase insulin secretion that played a major role in the reduction of HbA_1c_ although it occurred following the improvement of insulin sensitivity.

Our results showed that PIO also significantly increased APN levels in the whole and the obese group. This finding was consistent with other studies in obese populations [18, 19]. It was considered that the increase of APN reflected the improvement of tissue insulin resistance which is associated with increased cardiovascular disease (CVD) risk [14, 20] and heart failure [9]. In this regard, the increase of APN after the PIO therapy may be potentially effective in reducing CVD risk.

As for the adverse effects of PIO, we did not find significant body weight gain either in the obese group or in the lean group. Excitingly, some improvements in liver functions, blood pressure, and serum lipids profile were observed. Taken together with the reduction of HbA_1c_ and the increase of both insulin sensitivity and β-cell function, the results of this study clearly justified the efficacy and safety of PIO in both lean and obese Chinese type 2 diabetes patients.

## Limitation

Clinically, PIO is not a new antidiabetic agent, and its effect on plasma glucose reduction is well established. The main purpose of the present study was to confirm if PIO is effective in decreasing plasma glucose in both obese and non-obese type 2 diabetes patients, but not to compare the effectiveness between different groups. Therefore we did not use an untreated control group here. However, in order to avoid confounders, subjects who under some conditions may have experienced modified glucose metabolism were excluded, and all participants were required to retain their usual diet and lifestyle.

## Conclusion

A 16-week treatment of PIO significantly increased insulin sensitivity, first phase insulin secretion, and β-cell glucose sensitivity in the Chinese type 2 diabetes patients. A similar reduction of HbA_1c_ level in the lean and the obese group suggests that non-obese type 2 diabetic patients could also benefit from PIO treatment. The increase of circulating APN after the PIO treatment seems greater in the obese group than that in the lean group.
